# A Systematic Evaluation of Feature Selection and Classification Algorithms Using Simulated and Real miRNA Sequencing Data

**DOI:** 10.1155/2015/178572

**Published:** 2015-10-05

**Authors:** Sheng Yang, Li Guo, Fang Shao, Yang Zhao, Feng Chen

**Affiliations:** Department of Biostatistics, School of Public Health, Nanjing Medical University, 101 Longmian Road, Nanjing, Jiangsu 211166, China

## Abstract

Sequencing is widely used to discover associations between microRNAs (miRNAs) and diseases. However, the negative binomial distribution (NB) and high dimensionality of data obtained using sequencing can lead to low-power results and low reproducibility. Several statistical learning algorithms have been proposed to address sequencing data, and although evaluation of these methods is essential, such studies are relatively rare. The performance of seven feature selection (FS) algorithms, including baySeq, DESeq, edgeR, the rank sum test, lasso, particle swarm optimistic decision tree, and random forest (RF), was compared by simulation under different conditions based on the difference of the mean, the dispersion parameter of the NB, and the signal to noise ratio. Real data were used to evaluate the performance of RF, logistic regression, and support vector machine. Based on the simulation and real data, we discuss the behaviour of the FS and classification algorithms. The Apriori algorithm identified frequent item sets (mir-133a, mir-133b, mir-183, mir-937, and mir-96) from among the deregulated miRNAs of six datasets from The Cancer Genomics Atlas. Taking these findings altogether and considering computational memory requirements, we propose a strategy that combines edgeR and DESeq for large sample sizes.

## 1. Introduction

MicroRNAs (miRNAs) are small, endogenous, and noncoding RNAs that trigger messenger RNA (mRNA) deregulation and translational repression by binding the 3′ untranslated region (3′UTR) of these targets [1]. Depending on their biological function and stability, miRNAs are also regarded as biomarkers to distinguish cases and controls [2, 3]. Therefore, emerging technologies, such as cDNA microarrays, high-density oligonucleotide chips, and next-generation sequencing (NGS), have been highly useful in the discovery of miRNAs that cause or prevent disease [4]. cDNA microarrays and high-density oligonucleotide chips are only capable of providing relative expression levels, whereas NGS can be used to count the exact number of reads and obtain sequence information (arm switching and isomiRs) [5].

To process high-dimensional NGS data and gain deep insight into biological processes, statistical learning methods are emerging with the goal of classifying labels by selecting a subset of features, minimizing the coefficients of features or reducing their dimension [6, 7]. Using a negative binomial distribution (NB) assumption, edgeR, DESeq, and baySeq are three important filter algorithms for selecting significant variables by intrinsic characteristics [8–10]. Wrapper algorithms based on classification apply a search strategy in the feature space, including sequential forward searching (SFS) and sequential forward floating searching (SFFS); however, the computational intensity of this approach is large [11]. Hybrids of feature selection and classification, known as embedded methods, such as random forest (RF), regard the classification model as an internal parameter and reduce the computational requirements [12]. Furthermore, independent of the distribution, shrinkage tricks, such as lasso, also play an important role in high-dimensional NGS [13].

Recently, an evaluation of statistical and machine learning algorithms for NGS data has become essential. This evaluation can be achieved from three perspectives: (i) comparing the performance of seven popular feature selection algorithms in the context of simulation, using sensitivity and specificity; (ii) studying the properties of three classification algorithms, logistic regression, support vector machine (SVM), and RF, in the context of differentially expressed (DE) miRNAs from The Cancer Genomics Atlas (TCGA) data to gain deeper insight into the combination of FS and classification; and (iii) analysing the similarity of six cancers based on miRNAs and the corresponding pathways.

## 2. Methods

### 2.1. Simulations

First, we assumed that the distribution of NGS data was NB, corresponding to the parameters, mean, dispersion parameter (DP) of NB, and ratio of signal to noise (s2n) in the simulations. The inflating extent of the data is directly proportional to the DP, and s2n is the ratio of significant variables to insignificant variables. The second assumption was that all significant variables are causal, which indicates the means of case groups were larger than those of control groups.

Based on these two basic assumptions, three different settings were involved: s2n ranged from 0.01 to 0.2 (A1–A5), the means of the significant variables in the case group ranged from 10 to 30 by 5 (B1–B5), and the DP of the significant variables in the case group ranged from 0.125 to 8 (C1–C5). A total of 1,000 replications were produced to obtain a robust result. The parameter settings for the insignificant and significant variables were the same and fixed in all situations. When one parameter was studied, the others settings remained fixed. Details regarding the parameter settings are presented in Table 1.

### 2.2. Overviews of FS Algorithms and Their Evaluation Indexes

We compared seven different algorithms in the simulations, including three algorithms specific to NGS data (DESeq, edgeR, and baySeq), the Wilcoxon rank sum test, lasso, particle swarm optimal algorithm empowered by decision tree (PSODT), and RF. Each algorithm included different types of feature selection. The first five methods are filter methods because they select variables based on the order of the statistic or coefficient. PSODT, a wrapper algorithm, searches the subset of variables by PSO and evaluates the classification performance by DT. RF combines classification and feature selection. The Bioconductor packages* baySeq*,* DESeq2*, and* edgeR* were used, and lasso and RF were completed by the* glmnet* and* randomForest* packages in the R (version 3.0.3) framework, respectively.

DESeq and edgeR are two essential algorithms for feature selection in NGS data and are based on the NB distribution assumption. However, they use different methods for estimating the parameters. DESeq estimates the DP based on pooled data, which can normalize confounders from different library sizes. Local regression is then used to estimate the function of per-variable raw variance, a component of variance. edgeR algorithm defines the weighted conditional log-likelihood, which is a combination of common and individual likelihood, to estimate the parameter and uses *α* to weigh the importance of the common part. Exact testing is used by these two methods [14]. For baySeq, the difference between 1 and the posterior probability is considered as the *P* value. The* cv.glmnet* function estimates the penalty weight in lasso by cross-validation. We used the same parameter settings as Chen et al. for PSODT [11]. The score of each variable was identified as the time of* gbest* equal to* pbest*. For RF, we used the default setting, that is, the number of trees (*ntree*) = 500 and the number of random variables in each split (*mtry*) = $\sqrt{m}$, where *m* is the total number of variables.

In the simulations, type I errors and power were used to evaluate the performance of the four statistical algorithms (DESeq, edgeR, baySeq, and rank sum test) because they are based on hypothesis testing. Type I error and power correspond to the frequency of *P* values of noise and significant variables less than 0.05 or Bonferroni correction levels in 1,000 replications, respectively. As these procedures involved four machine learning methods, sensitivity and specificity were used to compare the entire techniques. These values were calculated according to

(1) Sensitivity=TPTP+FN,Specificity=TNTN+FB,

where TP, TN, FP, and FN are the means of the number of true cases, true controls, false cases, and false controls in 1,000 replications, respectively.

### 2.3. Real Data

For TCGA, six different cancer sequencing datasets (with features and samples) were involved, including breast invasive carcinoma (BRCA), head and neck squamous cell carcinoma (HNSC), kidney chromophobe (KICH), lung adenocarcinoma (LUAD), stomach adenocarcinoma (STAD), and thyroid carcinoma (THCA). We only selected the matched samples. The low expression miRNAs whose sum expression levels in all samples were less than 10 were excluded (Table 2).

### 2.4. Landscape of Classification Algorithms and Indexes

Classification algorithms, including logistic regression, RF, and SVM, were regarded as another essential point because they indicate the predictive performance of the selected biomarkers. Logistic regression, a type of generalized linear model (GLM), was widely applied in case-control study, as its exponential coefficient, odds ratio (OR), directly elucidated the risk of variables. Based on the theory of Lagrange duality and kernel function, SVM solved dual problems rather than the minimum primary problem and mapped the variables to a higher dimension. Therefore, the nonlinear classified samples were discriminated using hyperplane. The following equation shows the standard form of this method:

(2) $$\begin{array}{c} y=h_{w,b}\left(\;x\;\right)=w^{T}x+b. \end{array}$$

We chose the default settings of the* svm* function, which was a Gaussian kernel, and set the hyperparameter to *γ* = 3 and error term to *ε* = 0.2.

Random fivefold cross-validation was applied to real data to estimate the performance of the classification algorithms. This cross-validation meant that four-fifths of samples were used to construct the model and select the features, and the residual was used to test the validation; this process was replicated 100 times. The area under the ROC curve (AUC), positive predictive value (PPV), and negative predictive value (NPV) evaluated the classification performance of the featured subsets.

### 2.5. Apriori for Detecting the Frequent Item Set of miRNAs from Different Datasets

Apriori defines the frequency of item sets based on three indexes, including* support*,* confidence*, and* lift*. The* support* of an item set is defined as the percentage of the dataset that contained it. The* confidence* represents the association of the rule like {*A*} → {*B*}, which is calculated by the conditional probability of *P*(*B*∣*A*). The* lift*, the ratio of *P*(*B*∣*A*)/*P*(*B*), is the quotient of the posterior and the prior confidence of an association rule. The first two standards can select the frequent item set.

The frequent miRNA sets were defined from the DE miRNAs in the six datasets by the following criteria: (a) the miRNAs satisfied the Bonferroni correction; (b) the miRNAs were selected more than or equal to 80 times in one algorithm; and (c) the miRNAs were defined by at least 3 algorithms. The frequent DE miRNA was then identified as having* support* and* confidence* values larger than or equal to 0.5. Finally, their targets were predicted twice from three datasets (TargetScan, miRanda, and miRTarBase), and enrichment analysis defined the deregulated pathways by Gene Ontology (GO) [15–17].

## 3. Results

### 3.1. The Evaluation of FS Algorithms Using a Simulation

#### 3.1.1. Empirical Type I Error and Power of Four Statistical Algorithms

**Figure 1:**
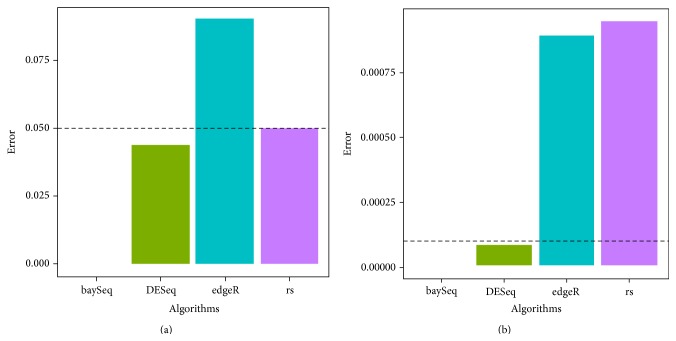
Type I error of four statistical algorithms. (a) *α* = 0.05 condition. (b) Bonferroni correction.

**Figure 2:**
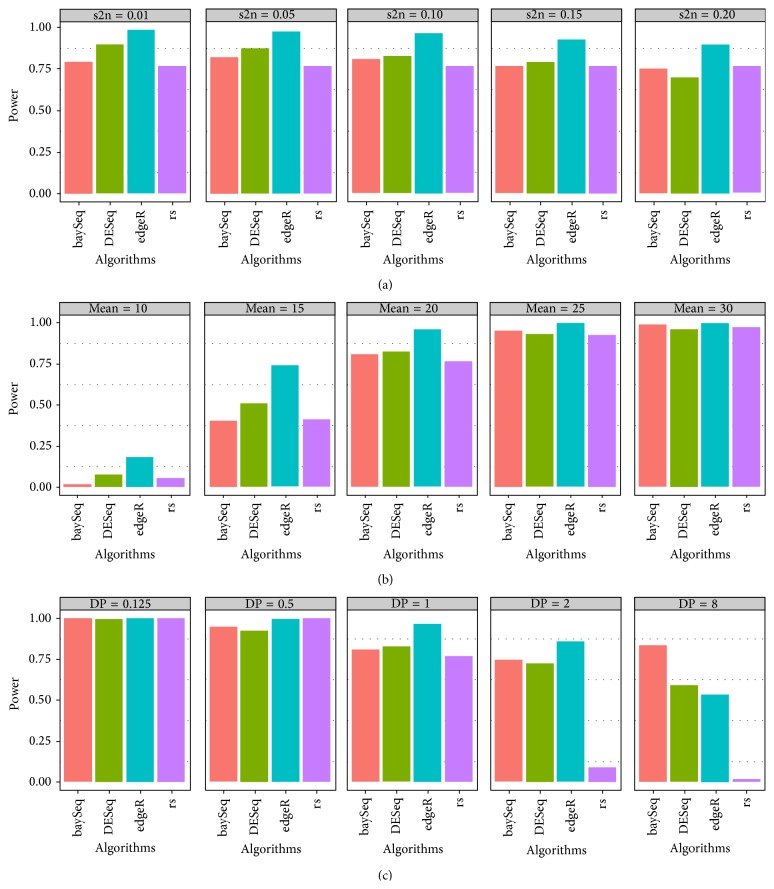
Power of four statistical algorithms with different settings of three parameters. (a) Different settings of the s2n of the variables. (b) Different settings of the mean. (c) Different settings of DP in the case group.

The type I error and power results are shown in Figures 1 and 2. baySeq, DESeq, and the rank sum test appeared to control type I error at a significance level of 0.05, although the rank sum test failed after Bonferroni correction. The type I error of edgeR was slightly inflated. s2n appeared to have no relationship to the power, whereas the mean and DP influenced the power. Based on the difference between the increasing mean or decreasing DP, the power of all of the algorithms increased. In particular, a decreasing trend in the rank sum test was observed with increasing DP because it included little consideration of the dispersion of the variables. However, the power of the three sequencing methods was high, especially for baySeq.

#### 3.1.2. Sensitivity and Specificity with Different Settings of Three Parameters

**Figure 3:**
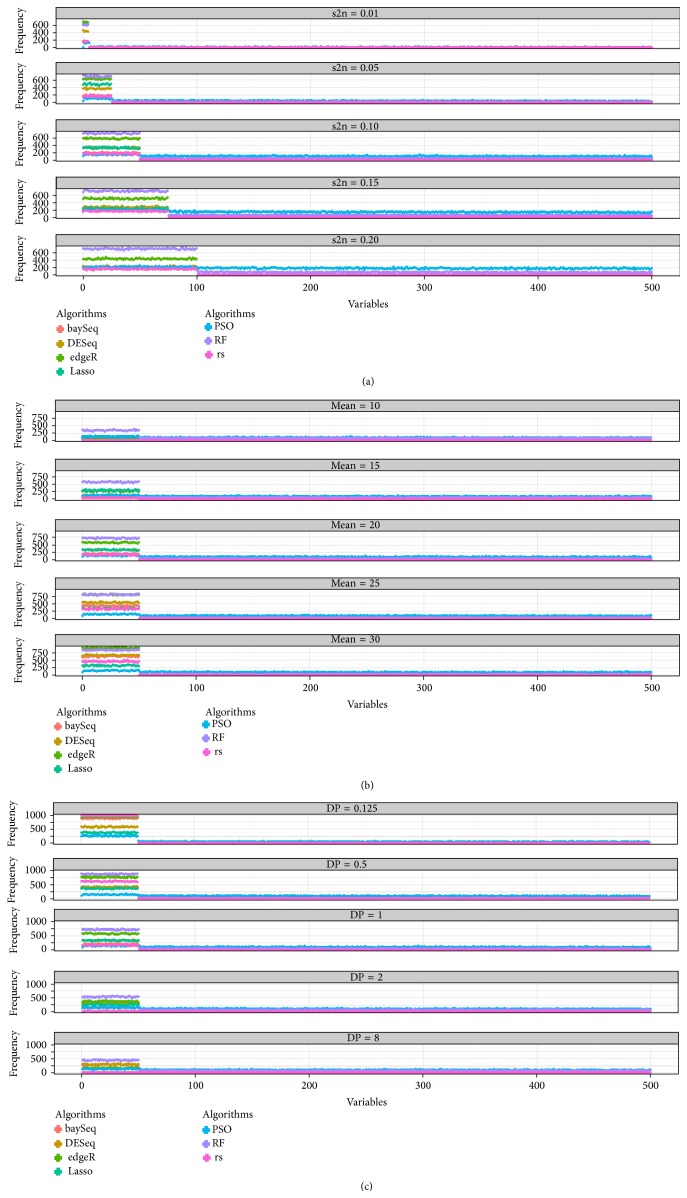
The frequency of selected variables of seven FS methods in the simulation. (a) Different settings of the s2n of the variables. (b) Different settings of the mean. (c) Different settings of DP.

The results from the simulation using scenarios A1–A5, B1–B5, and C1–C5, including the variable frequency, sensitivity, and specificity in different situations, are presented in Table 3 and Figure 3. First, DP influenced the two indexes of the machine learning algorithms and rank sum, although it had only a small influence on the performance of three sequencing methods. The sensitivities of edgeR and DESeq were larger than that of baySeq, although the extent of the increase and decrease of their sensitivity was larger. With increasing dispersion, the sensitivities of the rank sum and lasso methods were approximately zero. Second, when the difference between the means of the case and the control samples increased from 5 to 25, the sensitivity increased to different extents. For the three sequencing methods and the rank sum test, the index showed an obvious increase, and the frequency of selected significant variables was higher. The sensitivity and specificity of PSODT and RF showed little change with the change of mean. Third, greater s2n values led to increased sensitivity for the frequency of significant variables of baySeq and RF but appeared to have no relationship with the residuals.

For the seven algorithms, we obtained the following outcomes. The sensitivity of baySeq appeared to be lower than the other sequencing methods. The variations of the power of DESeq and edgeR were relatively similar, although the latter was not control type I error. Lasso also strictly controlled type I errors, although its power was lower than that of the other methods in multiple situations. The rank sum test, a nonparametric method, was also influenced by the three parameters and is perhaps not suitable for sequencing data. In particular, when DP increased from 0.125 to 8, its sensitivity decreased from 1.00 to 0.23. The sensitivity of PSODT was highly stable when the parameters changed. The sensitivity of RF was only related to the s2n factor.

### 3.2. The FS and Classification Methods in Real Data

**Figure 4:**
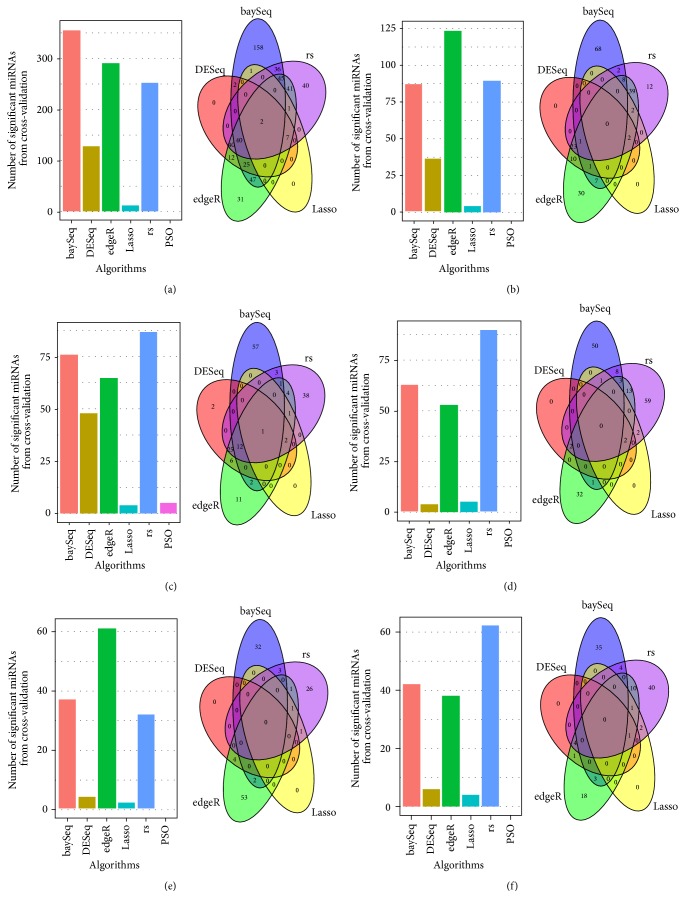
The bar plots and Venn diagrams of a number of significant miRNAs identified by different FS algorithms in six cancers. The bar plot indicates the number of significant variables. The Venn diagram illustrates the relationships of the significant variables among the six methods. (a) BRCA; (b) HNSC; (c) KICH; (d) LUAD; (e) STAD; and (f) THCA.

The number of significant miRNAs identified by different FS algorithms and the relationships between them are shown in Figure 4 and Additional File 1 available online at http://dx.doi.org/10.1155/2015/178572. Based on the frequency bar plots and Venn diagrams of each dataset, these results are clear. First, baySeq, edgeR, and the rank sum test selected the highest number of miRNAs in different datasets. For example, in KICH, the rank sum test selected 87 significant miRNAs, which was the greatest number of significant miRNAs identified by the six algorithms. Second, the three sequencing methods and the rank sum test had more intersections. However, PSODT rarely identified the same significant miRNAs during cross-validation, and intersections were also rare.

As shown in Table 4, the results of classification algorithms were as follows. First, RF and SVM performed better than logistic regression. For example, based on the results from edgeR in the KICH, the ROC of logistic regression was 0.39, which was lower than that of RF and SVM. Interestingly, logistic regression performed best using the variables selected by lasso, perhaps because the ratio between the number of variables and the number of samples was unsuitable for logistic regression, with the exception of lasso. Second, although the power of PSODT was lowest among the seven FS algorithms, the classification performance was not the worst. For example, in BRCA, the classification of the variables selected form PSODT was better than that of the rank sum test.

### 3.3. Run Time

The run time of the seven algorithms is shown in Additional File 2. In the simulations, baySeq required approximately 2 hours, which is longer than the other methods. However, different results were observed using real data. The time of DESeq sharply increased with larger sample sizes; however, the variations of other methods were not obvious with increasing sample sizes. Thus, baySeq consumed the greatest computational resources, and the resource consumption of DESeq in particular was largely determined by the sample size.

### 3.4. The Frequency miRNA Sets in Six Cancers and Enrichment Analysis

For the DE miRNAs in each cancer set, Apriori selected the frequency item sets that might be co-DE miRNAs in cancers (Additional File 3). mir-133a-1, mir-133b, mir-183, mir-937, and mir-96 were frequently identified DE miRNAs in six cancers. Some miRNAs were deregulated at the same time; for example, the* confidence* of mir-96 to mir-133a-1 was 1, and the* lift* was equal to 2. Furthermore, the enrichment pathways of their cotargets were also identified using GO (Additional Files 4 and 5).

## 4. Discussion

Using simulations and real data, we compared the performance of seven feature selection algorithms and three classification algorithms. Simulations identified the differing performances of the seven FS methods: baySeq, DESeq, edgeR, the rank sum test, lasso, PSODT, and RF. In the comparisons of four statistical methods, we observed the following: (a) a larger DP may lead to a low power in the rank sum test due to a failure to estimate DP; (b) when the difference of the mean is greater than 15, the power of the sequencing methods is robust; (c) with increasing DP, there is a small decrease in the power of the sequencing methods, especially for baySeq. Regarding the sensitivity and specificity, the following conclusions were reached: (a) s2n influences the performance of baySeq and RF; (b) an increase in the difference of means causes increased sensitivity; and (c) increasing DP has little effect on the three sequencing algorithms but decreases the sensitivity of the others. Furthermore, real data showed that (a) logistic regression is unsuitable for the high dimension and small sample data and (b) the performance of RF is better than that of SVM.

Moreover, seven algorithms were evaluated using different conditions. edgeR was found to be suitable for large sample sizes because of low calculation time, although its type I error increases slightly. The type I error and power indicate that the performance of baySeq is perhaps best for selecting significant genes, although a large sample size may require a long computation time [18]. Similar to baySeq, DESeq requires more time with increasing sample size, although its advantage is that it can analyse data using only one replicate in each treatment group (Figure 1 and Additional File 2) [10]. The selection of the three algorithms is determined by the experimental design [18]. The rank sum test can be fit to any distribution assumption, but it fails to select the variables in NB, especially with increasing DP. The penalty of lasso is possibly too large because few significant variables are selected. PSODT rarely chooses the significant variables and has no association with the three factors because it defines a combination of variables having the best performance of DT. Considering the power, type I error, and calculation cost, an FS selection process can consist of two or more processes: (a) primary selection, which requires fast and high-power algorithms, and (b) further selection, which requires an algorithm that controls type I error. In our study, we present the combination of edgeR and DESeq as a strategy for selecting the significant variables for large sample sizes.

This study has some advantages over previous studies [18, 19]. First, the simulations not only assumed that the NGS data had a NB distribution but also compared the FS or classification algorithms in different settings of the mean, DP, and s2n. Lacking a gold standard, the real data failed to compare the FS methods. To guarantee the effectiveness, the parameter settings are obtained from the real data. Second, this study involves not only three sequencing algorithms but also machine learning methods.

However, this study also has many drawbacks. First, the three involved classifiers perhaps neglect the interactions between different variables; however, the interactions play important roles in explaining the association between molecules and diseases. With the network successfully used in biology, the classifiers based on network are perhaps more suitable to explain the association [20]. Second, some new bioinformatics classifiers are not included, such as LibD3C, HPFP, and miRClassify [21–23]. Particularly, LibD3C, classifying the cytokines from the protein sequence, applies ensemble classifiers in each layer to improve the prediction accuracy and uses SMOTE to overcome the imbalance of samples. It also selects 120 features as the eight physicochemical properties of protein and can be used in analyzing the sequencing data [21].

When studying real data, we found that mir-133a-1, mir-133b, mir-183, mir-937, and mir-96 were frequent miRNAs sets in six cancers, and some combination of these can increase the probability of finding others. By regulating the expression of MCL-1 and BCL2L2, mir-133b is associated with lung cancer, which was also observed in our results [24]. As one of the frequent item sets, mir-133b is also related to oesophageal squamous cell carcinoma by* FSCN1* [25]. mir-96 and mir-183 both contribute to the stage and grade of urothelial carcinoma [26].

In conclusion, we propose the use of a combination of edgeR and DESeq to analyse miRNA sequencing data with a large sample size. Apriori detects the frequent item sets that might contribute to other tumours.

## Supplementary Material

File 1-2: Two files show the similarity and the computational consume of the six feature selection methods, respectively.
File 3-5: The three tables are the results of the principle steps of miRNA analysis, including detecting DE miRNAs, predicting their targets and enrichment analysis. The three steps are from selection miRNAs to biological function prediction. File 3 shows the three indexes of the elements of frequent term set. File 4 lists the predicted targets of them form three datasets. File 5 list the top pathways from enrichment analysis.

## Figures and Tables

**Table 1:** Parameter settings used for the simulation data.

Scenario	Parameter	Settings
A1–A5	Signal to noise (s2n)	0.01, 0.05, 0.1, 0.15, and 0.20
B1–B5	Mean of significant variables in the case	10, 15, 20, 25, and 30
C1–C5	Dispersion parameter of significant variables in the case	0.125, 0.5, 1, 2, and 8
	Sample size (+/−)	40 (20/20)
	Number of variables	500
	Mean of significant variables in the control	5
	Mean of insignificant variables	5
	Dispersion parameter of significant variables in the control	1
	Dispersion parameter of insignificant variables	1

**Table 2:** Summary of the selected datasets.

Number	Cancer	Feature	Sample (+/−)	SDR^a^
1	BRCA	903	206 (103/103)	0.23
2	HNSC	906	162 (81/81)	0.18
3	KICH	796	82 (41/41)	0.10
4	LUAD	895	218 (109/109)	0.24
5	STAD	857	170 (85/85)	0.20
6	THCA	904	212 (106/106)	0.23

^a^SDR refers to the ratio between the number of samples and the number of features.

**Table 3:** Sensitivity and specificity of the seven algorithms in different settings^a^.

Scenario	baySeq		DESeq		edgeR		Lasso	Rank sum		PSODT	RF
	*P* = 0.05	Bon^b^	*P* = 0.05	Bon^b^	*P* = 0.05	Bon^b^		*P* = 0.05	Bon^b^		
s2n											
A1	0.68/1.00	0.16/1.00	0.97/0.96	0.43/1.00	0.99/0.93	0.68/1.00	0.62/0.99	0.92/0.95	0.16/1.00	0.10/0.99	0.60/1.00
A2	0.74/1.00	0.18/1.00	0.97/0.96	0.37/1.00	0.98/0.92	0.63/1.00	0.49/0.99	0.92/0.95	0.16/1.00	0.11/0.95	0.70/0.98
A3	0.77/1.00	0.19/1.00	0.95/0.94	0.32/1.00	0.98/0.91	0.57/1.00	0.32/1.00	0.92/0.95	0.16/1.00	0.14/0.90	0.71/0.97
A4	0.77/1.00	0.17/1.00	0.93/0.93	0.27/1.00	0.96/0.89	0.50/1.00	0.23/1.00	0.92/0.95	0.16/1.00	0.18/0.86	0.70/0.95
A5	0.76/1.00	0.16/1.00	0.90/0.90	0.22/1.00	0.95/0.86	0.43/1.00	0.18/1.00	0.18/1.00	0.92/0.95	0.17/1.00	0.70/0.93

Mean of significant variables											
B1	0.05/1.00	0.00/1.00	0.41/0.95	0.01/1.00	0.52/0.92	0.04/1.00	0.13/0.99	0.40/0.95	0.01/1.00	0.12/0.90	0.33/0.93
B2	0.45/1.00	0.03/1.00	0.81/0.95	0.12/1.00	0.88/0.92	0.27/1.00	0.30/1.00	0.77/0.95	0.05/1.00	0.13/0.90	0.57/0.95
B4	0.91/1.00	0.41/1.00	0.99/0.94	0.53/1.00	0.99/0.91	0.78/1.00	0.32/1.00	0.97/0.95	0.31/1.00	0.14/0.91	0.79/0.98
B5	0.96/1.00	0.62/1.00	1.00/0.94	0.66/1.00	1.00/0.90	0.90/1.00	0.32/1.00	0.99/0.95	0.45/1.00	0.14/0.91	0.83/0.98

Dispersion parameter of significant variables											
C1	1.00/1.00	0.87/1.00	1.00/0.92	0.57/1.00	1.00/0.89	0.92/1.00	0.37/1.00	1.00/0.95	0.97/1.00	0.26/0.95	0.97/1.00
C2	0.89/0.99	0.38/0.94	0.98/1.00	0.40/0.94	0.99/1.00	0.75/0.97	0.35/0.93	1.00/1.00	0.61/0.96	0.14/0.90	0.86/0.90
C4	0.71/1.00	0.14/1.00	0.90/0.95	0.29/1.00	0.92/0.92	0.36/1.00	0.24/0.99	0.46/0.95	0.01/1.00	0.14/0.90	0.52/0.95
C5	0.73/1.00	0.28/1.00	0.73/0.96	0.29/1.00	0.71/0.93	0.16/1.00	0.00/1.00	0.23/0.95	0.00/1.00	0.13/0.90	0.44/0.94

^a^The conditions where the mean = 20, dispersion parameter = 1, and s2n = 0.1 are the same. Each cell includes the sensitivity and specificity.

^b^Bon indicates a result using the Bonferroni correction.

**Table 4:** Summary of three classification methods using real data.

Datasets	FS	Logistic regression			RF			SVM		
		PPV	NPV	AUC	PPV	NPV	AUC	PPV	NPV	AUC
BRCA	baySeq	0.53	0.53	0.53	1.00	0.99	0.99	0.95	0.96	0.96
	DESeq	0.70	0.72	0.70	1.00	0.99	1.00	1.00	0.94	0.97
	edgeR	0.54	0.55	0.55	1.00	0.99	0.99	0.54	0.55	0.55
	Lasso	0.97	0.98	0.98	1.00	0.99	0.99	0.97	0.98	0.98
	Rank sum	0.55	0.55	0.55	1.00	0.99	0.99	0.55	0.55	0.55
	PSODT	0.85	0.86	0.86	0.99	0.98	0.98	0.85	0.86	0.86

HNSC	baySeq	0.35	0.38	0.37	0.54	0.56	0.55	0.63	0.52	0.58
	DESeq	0.52	0.57	0.55	0.53	0.52	0.52	0.91	0.47	0.69
	edgeR	0.32	0.35	0.33	0.54	0.54	0.54	0.32	0.35	0.33
	Lasso	0.52	0.76	0.64	0.55	0.55	0.55	0.52	0.76	0.64
	Rank sum	0.35	0.31	0.33	0.54	0.54	0.54	0.35	0.31	0.33
	PSODT	0.43	0.44	0.43	0.55	0.54	0.54	0.43	0.44	0.43

KICH	baySeq	0.36	0.38	0.37	0.65	0.66	0.66	0.68	0.70	0.69
	DESeq	0.37	0.39	0.38	0.66	0.65	0.66	0.68	0.84	0.76
	edgeR	0.40	0.38	0.39	0.66	0.65	0.66	0.40	0.38	0.39
	Lasso	0.64	0.82	0.73	0.65	0.66	0.65	0.64	0.82	0.73
	Rank sum	0.39	0.38	0.39	0.66	0.66	0.66	0.39	0.38	0.39
	PSODT	0.37	0.38	0.37	0.66	0.65	0.66	0.37	0.38	0.37

LUAD	baySeq	0.40	0.47	0.43	0.46	0.45	0.46	0.45	0.69	0.57
	DESeq	0.30	0.78	0.54	0.46	0.41	0.44	0.95	0.36	0.65
	edgeR	0.44	0.47	0.46	0.47	0.45	0.46	0.44	0.47	0.46
	Lasso	0.47	0.74	0.61	0.47	0.45	0.46	0.47	0.74	0.61
	Rank sum	0.30	0.36	0.33	0.47	0.45	0.46	0.30	0.36	0.33
	PSODT	0.36	0.50	0.43	0.47	0.45	0.46	0.36	0.50	0.43

STAD	baySeq	0.42	0.56	0.49	0.44	0.45	0.44	0.44	0.63	0.54
	DESeq	0.14	0.85	0.49	0.41	0.38	0.40	0.91	0.25	0.58
	edgeR	0.37	0.42	0.40	0.49	0.46	0.47	0.37	0.42	0.40
	Lasso	0.43	0.77	0.60	0.46	0.46	0.46	0.43	0.77	0.60
	Rank sum	0.40	0.48	0.44	0.44	0.46	0.45	0.40	0.48	0.44
	PSODT	0.36	0.44	0.44	0.44	0.46	0.45	0.36	0.44	0.40

THCA	baySeq	0.49	0.63	0.56	0.56	0.57	0.57	0.77	0.50	0.63
	DESeq	0.49	0.85	0.67	0.54	0.58	0.56	0.54	0.82	0.68
	edgeR	0.53	0.59	0.56	0.56	0.60	0.58	0.53	0.59	0.56
	Lasso	0.54	0.88	0.71	0.56	0.59	0.57	0.54	0.88	0.71
	Rank sum	0.44	0.44	0.44	0.57	0.58	0.58	0.44	0.44	0.44
	PSODT	0.48	0.56	0.52	0.56	0.56	0.56	0.48	0.56	0.52
